# The impact of Cenozoic cooling on assemblage diversity in planktonic foraminifera

**DOI:** 10.1098/rstb.2015.0224

**Published:** 2016-04-05

**Authors:** Isabel S. Fenton, Paul N. Pearson, Tom Dunkley Jones, Alexander Farnsworth, Daniel J. Lunt, Paul Markwick, Andy Purvis

**Affiliations:** 1 Department of Life Sciences, Natural History Museum, Cromwell Road, London SW7 5BD, UK; 2 Department of Life Sciences, Imperial College London, Silwood Park Campus, Ascot SL5 7PY, UK; 3 School of Earth and Ocean Sciences, Cardiff University, Cardiff CF10 3AT, UK; 4 School of Geography, Earth and Environmental Sciences, University of Birmingham, Edgbaston, Birmingham B15 2TT, UK; 5 School of Geographical Sciences and Cabot Institute, University of Bristol, Bristol BS8 1SS, UK; 6 Getech Group plc. Elmete Hall, Elmete Lane, Leeds LS8 2LJ, UK

**Keywords:** planktonic foraminifera, Eocene, latitudinal diversity gradient, global climate model

## Abstract

The Cenozoic planktonic foraminifera (PF) (calcareous zooplankton) have arguably the most detailed fossil record of any group. The quality of this record allows models of environmental controls on macroecology, developed for Recent assemblages, to be tested on intervals with profoundly different climatic conditions. These analyses shed light on the role of long-term global cooling in establishing the modern latitudinal diversity gradient (LDG)—one of the most powerful generalizations in biogeography and macroecology. Here, we test the transferability of environment-diversity models developed for modern PF assemblages to the Eocene epoch (approx. 56–34 Ma), a time of pronounced global warmth. Environmental variables from global climate models are combined with Recent environment–diversity models to predict Eocene richness gradients, which are then compared with observed patterns. The results indicate the modern LDG—lower richness towards the poles—developed through the Eocene. Three possible causes are suggested for the mismatch between statistical model predictions and data in the Early Eocene: the environmental estimates are inaccurate, the statistical model misses a relevant variable, or the intercorrelations among facets of diversity—e.g. richness, evenness, functional diversity—have changed over geological time. By the Late Eocene, environment–diversity relationships were much more similar to those found today.

## 1. Introduction

Ecologists and palaeontologists have long been interested in the causes of diversity patterns found in many taxonomic groups, but the two communities often approach such questions in different ways [1,2]. Ecologists are able to obtain diversity estimates across the globe, but usually lack a temporal perspective (e.g. [3]), whereas palaeontologists typically have a more limited spatial resolution at any given point in time [4]. Each approach has its strengths and limitations. If evolutionary processes such as speciation, immigration and extinction have been important for structuring Recent diversity patterns (e.g. [5,6]), then incorporating fossil data into studies of these patterns is likely to be important [7]. Although phylogenies of extant species contain some information about the rates of these processes, they lack direct information about ancestors or extinct species limiting the inferences that can be drawn [8,9]. Combining ecological and palaeontological approaches is likely to aid our understanding of diversity patterns [1,2,10,11].

The latitudinal diversity gradient (LDG) is one of the most widely studied of all diversity patterns. It occurs in a very broad range of habitats and taxa [12,13]. Despite being well described, there is little consensus on whether the LDG has one dominant underlying cause or reflects multiple causes acting in concert [12,14,15]. Community species richness is often found to be correlated with environmental variables (e.g. [15,16]), but these correlations do not prove that the true causes have been identified [17]. Instead, these correlations could have a separate, underlying cause. Using an independent dataset can improve support for the hypothesis that the important variables have indeed been captured.

There are three ways to obtain such an independent dataset. The first is to test the predictions in a different region. This is not possible for global diversity studies where the whole clade is included in the original analysis. The second method transfers the model to a different clade (e.g. [18–20]). There are, however, limits to how well any model would be expected to transfer among clades, given that all clades have some degree of difference in ecological traits and tolerances. It is thus hard to separate a mismatch caused by ecological differences from that caused by an imperfect model. The third method transfers the model to a time period with substantially different environmental conditions, a concept often used in global climate modelling (e.g. [21]). Typically, macroecologists lack a deep-time temporal perspective, limiting such transfers to a few decades (e.g. [22,23]). Such short timescales are unlikely to be informative for global diversity patterns; global environmental conditions will not vary sufficiently and they are likely to be too short for macroevolutionary processes such as speciation to act.

Most study systems, even those with excellent fossil records, are so spatio-temporally and/or taxonomically incomplete that advanced methods are needed to estimate even simple diversity measures such as species richness [24] and biogeographic history [25]. Without the use of such methods, spatio-temporal diversity patterns derived from the fossil record of most extant clades are too fragmentary for a robust test of hypotheses developed from the modern biota. Two approaches have been used to obtain sufficient species-level data for diversity gradient studies: (i) the study of a small number of sites with very high-quality data are compared to a known diversity gradient, e.g. that in the Recent (e.g. [26]); or (ii) all known fossils from a time period are grouped into latitudinal bins (e.g. [27]).

The planktonic foraminifera (PF) provide a rare study system where neither approximations nor complex methods are necessary: assemblage diversity estimates can be obtained consistently across large spatial and temporal scales. As such their importance for deepening our understanding of macroecological patterns is now being recognized [2,16]. These calcareous open-ocean protists have the most complete fossil record of any clade [28,29], with species having at least an 81% chance of being recorded from any given 1 Myr time bin [30], equivalent to the best genus-level completeness for macroinvertebrates [31]. Deposition in deep marine environments, where PF are typically recorded, is more continuous than either shallow marine or terrestrial environments [32], reducing the need to untangle the correlation between rock record and diversity that occurs in the other settings (e.g. [33,34]). Assemblages from the deep-ocean are thus more equivalent across spatial and temporal scales than terrestrial or shallow marine assemblages [35].

In this paper, we start by summarizing the geographic patterns of Recent PF diversity and the macroecological models used to determine the drivers of that diversity. We then make use of the clade's exceptional fossil record, interrogating a new collation of Eocene assemblage data, to determine diversity patterns in the Eocene. We combine our present day statistical models with palaeoenvironmental data from climate models to derive estimates of Eocene diversity patterns which are then compared to observed Eocene diversity to determine the transferability of Recent macroecological models.

## 2. Recent planktonic foraminiferal diversity

Foraminifera are unicellular zooplankton with a test or ‘shell’, usually made of calcium carbonate. This test consists of a series of chambers which are added progressively as the organism grows; holes, or foramina, allow movement of cytoplasm between these chambers [36]. Foraminifera show two main lifestyles: benthic foraminifera live on the seafloor, while PF float passively in the ocean. PF can be further split into macroperforates or microperforates, depending on the size of the pores in the test. Here, we focus on macroperforate species, which are less susceptible to dissolution than the microperforates, are more frequently identified in palaeontological studies, comprise a single and dominant clade, and have a better understood taxonomy and ecology [29].

The tests of fossil PF include all the taxonomically diagnostic morphological characters used to classify living specimens. This makes Recent and fossil PF morphospecies fully comparable, contrasting with the situation in most other taxa where non-comparability of taxonomic categories hampers comparisons of fossil and Recent diversity [37]. Molecular analyses (using small subunit (SSU) rRNA) however suggest some extant morphospecies should be split into cryptic or semi-cryptic genetic species, with slightly different ecological and biogeographical preferences (e.g. [28,38,39]). To date, around half of modern morphospecies have been sequenced, with the presence of cryptic diversity varying across the clade [39]. For example, genetic studies of *Orbulina universa* identified three pseudo-cryptic species inhabiting different hydrological conditions [40], whereas the *Trilobatus sacculifer* complex contains a wide range of morphologies while showing no evidence for genetic variability [41,42]. As possible cryptic species within a morphospecies are mostly geographically separated, occupying slightly different ecological or environmental niches (e.g. [39]), they are likely to have more impact on global diversity estimates than on species richness counts at a given location. It is also important to note the cryptic species problem is not unique to PF, and the ratio of possible genetic species to morphospecies is in line with that seen in other taxa (e.g. [43]). Although SSU rRNA underestimates true diversity in many other groups [44], it evolves much more rapidly in PF [45].

PF are well sampled both in the Recent and through the Cenozoic, as a result of their use as stratigraphic marker fossils and for palaeoenvironmental proxies [46]. Core-top marine sediment samples are routinely analysed for oceanographic studies, including collecting census data for calcareous microplankton. Sample preparation and data collection are usually broadly consistent between studies, in marked contrast to much terrestrial biodiversity monitoring. Typically, the sample is sieved at 150 µm and 300 individuals are counted (following [47]). The MARGO dataset, used to provide the Recent diversity data in this analysis, contains approximately 4000 assemblage counts of Recent core-top samples ([48]; electronic supplementary material, figure S1). The data include relative abundances of all observed species; only morphospecies that are genuinely absent, very rare or very small (and so passing through the sieve) will not be recorded in such an assemblage count.

**Figure 1.**
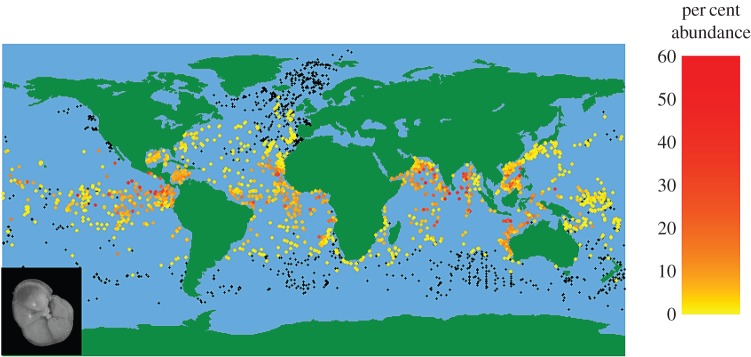
Percentage abundance of *Menardella menardii* in the MARGO dataset; black points indicate absence.

These data can be visualized as species distribution maps (e.g. figure 1). Based on these distributions, extant PF diversity is split into six faunal provinces each with a distinct community [36,49]. Few species are found exclusively in one faunal province; instead such provinces are defined by the dominant species. Provinces tend to correspond to the major ocean gyre systems, running roughly parallel to lines of latitude. High-productivity upwelling regions, however, are distinct from other sites at the same latitude. In the modern oceans, these regions tend to contain relatively higher abundances of *Neogloboquadrina*, *Globigerina bulloides* and some genetic variants of *Globigerinella siphonifera* [39].

Assemblage count data have long been used to calculate a wide range of site-level diversity measures based on taxonomic diversity [50]. Data compilations of species' traits and evolutionary relationships [29] now also allow functional and phylogenetic diversity to be calculated. Recent developments in automating trait measurements allow studies in the spatial patterns of morphological variance [51]. As these core-top samples represent death assemblages, they are not directly comparable to a single census of live specimens. Communities living at different depths or in different seasons are averaged in the sediment. Lateral transport of individuals before deposition may also lead to a certain amount of spatial averaging [52], but this is thought to be relatively minimal for PF [28].

Here, we focus on four diversity measures to characterize different aspects of assemblage diversity: rarefied species richness, Simpson's evenness, mean evolutionary age (MEA) and functional richness. Rarefied species richness is a sample-size-independent estimate of site-level species richness [53]; samples were rarefied to 275 individuals. Evenness quantifies whether assemblages are dominated by a small number of species. To remove the correlation with species richness which can occur in evenness measures (e.g. Shannon diversity, Simpson's index), we calculate Simpson's evenness calculated as Simpson's index divided by species richness: low values imply a few species dominate [54]. Functional richness is defined as the amount of functional space filled by the community and measured using Villéger *et al.*'s [55] FRic metric. The functional traits included in this measure are the test structure and size, as well as life habits of the species (presence/absence of spines and symbionts, ecogroup and morphogroup [29], depth habitat, dissolution susceptibility). The MEA is the (abundance-weighted) average age of the species lineages present in an assemblage [56]. To calculate this measure accurately requires a fossil record of the quality of the PF. Molecular phylogenies will always assign sister species the same age, defining age as the split between two extant species [57]. Morphospecies ages in the fossil record can also be biased: PF morphospecies are used as zone fossils, encouraging the fine-scale division of species by time [46], even in the absence of cladogenetic events, meaning that the appearance of a morphospecies may reflect a pseudospeciation (e.g. [58,59]). We therefore use evolutionary species (lineages), as determined by Aze *et al.* [29], for calculating the ages. These originate at cladogenesis and terminate with extinction of the lineage, but can persist through speciation if the speciation has a budding (offshoot) rather than bifurcation (equal splitting) pattern [60–62]. A younger MEA implies that species in an assemblage have arisen more recently. However, a community's MEA does not imply that it has existed for that length of time, or that any speciation occurred *in situ.*

The latitudinal variation of these four diversity measures for the Recent PF is explored in detail by Fenton *et al.* [56]. Whereas the peak in terrestrial species richness typically occurs at the Equator [12], PF—in common with many other marine taxa [13,63]—have two peaks in diversity at about ±20° latitude. These peaks correspond to the oligotrophic subtropical gyres, whereas the Equator is subject to upwelling of deep, nutrient-rich water, creating more eutrophic conditions. Functional diversity, like richness, is low at high latitudes; but it approaches its maximum more rapidly, showing little variation below 40°. This levelling out implies functional redundancy in tropical regions. Temperate and high-latitude sites typically have more equal abundances than tropical sites, i.e. have a higher Simpson's evenness. Upwelling regions are less speciose, with less even assemblages. Although MEA shows relatively little change with latitude, the oldest assemblages are in subpolar waters while polar assemblages are the youngest (but are very species-poor).

### (a) Drivers of Recent diversity

Explanations of diversity patterns can be split into ecological, evolutionary, historical or statistical causes (e.g. [64,65]). Ecological causes assume that the LDG results from processes acting over relatively short spatial and temporal scales, on factors such as suitable habitat, available energy, competition between species and dispersal limitations. For example, Briggs [66] suggests richness is higher at warmer temperatures as there is sufficient energy for specialization on relatively low-energy food sources, which at cooler temperatures would not suffice. Evolutionary explanations for the LDG invoke different rates of speciation, extinction or immigration at different latitudes (e.g. [5,66]). The ‘out of the tropics’ model, for example, assumes that taxa preferentially originate in the tropics, subsequently expanding into higher latitudes. The metabolic theory of ecology relates high tropical richness to faster speciation at higher temperatures [67]. Historical explanations implicate a region's history through geological time—either changes in the physical properties, such as plate tectonics driving climate change, or the occurrence of contingent events, such as meteorite impacts. The Mid-Domain Effect (MDE), a statistical explanation, states that if species' ranges are placed randomly into an area with hard boundaries, a gradient will develop with a central peak. The width of this peak depends on range sizes, being wider when ranges are small. As the poles act as hard boundaries, the MDE will produce an equatorial peak in diversity, i.e. an LDG [68]. Alternative versions of the MDE suggest that it could act on an environmental gradient such as temperature [20].

Macroecological models of foraminiferal diversity have mostly focused on species richness, often in the Atlantic Ocean. Rutherford *et al.* [16] reported nearly 90% of species richness variation in the Atlantic can be explained by sea surface temperature (SST); other variables, such as temperature at depth, productivity and salinity, did not significantly improve their models (table 1). They suggested that SST influences diversity by controlling the vertical partitioning of the water column, with the associated creation of distinct niches, but did not test this hypothesis. A similar study [13] of multiple taxa and all the oceans found multiple factors were independently significant predictors of richness (table 1), although temperature was still the most important. The correlation between species richness and temperature has been found to hold for the last 3 Myr [71]

**Table 1.** Variables used in studies of global diversity in Recent PF. 1, Rutherford *et al.* [16], used polynomial regressions; 2, Brayard *et al.* [69], used a bioclimatic model; 3, Morey *et al.* [70], used a Canonical Correspondence Analysis; 4, Tittensor *et al.* [13], used spatial autoregressive models; 5, Beaugrand *et al.* [20], used a bioclimatic model; 6, Fenton *et al.* [56], used spatial autoregressive models; 7, this study.

category	variable	effect	study
energy input	mean annual SST	mid-temperature peak	1, 3, 4, 6, 7
	annual solar irradiance	positive	1
	MDE on SST	significant	2, 5
vertical temperature structure	mixed-layer depth	mid-depth peak	1, 6, 7
	mixed-layer depth variation	none	1
	10°C depth	mid-depth peak	6, 7
	temperature at 150 m	mid-temperature peak	1
seasonal assemblages	SST variation	none	1, 3, 4, 6, 7
	salinity variation	negative	6, 7
productivity	mean log productivity	mid-productivity peak/none	3, 4, 6
	mean annual chlorophyll-a	significant	1, 3
	1% light depth	none	1
	dissolved nitrate	significant	1, 3
	phosphate	significant	3
stress	mean salinity	mid-salinity peak	1, 3, 6, 7
	oxygen stress	negative	1, 4, 6
ocean currents	mean annual topography	significant	1
	mean geostrophic current velocity	none	1
	SST slope	positive	4
geography	ocean	none/significant	4, 6
	coastline length	negative	4
	water depth	significant	3
ecological	temperature niche breadth	significant	5
evolution	geographical origin	significant	2
other	dissolution (when sites with significant dissolution are removed)	none	3, 6, 7
	density	significant	3

It is becoming increasingly recognized that incorporating multiple facets of diversity (e.g. taxonomic, phylogenetic, functional) improves understanding of the origins of diversity patterns [72,73]. Other studies have related more aspects of community structure to a set of environmental variables (table 1). Morey *et al.* [70] used canonical correspondence analysis to identify environmental correlates of community structure but did not interpret their results in terms of drivers of diversity. Fenton *et al.* [56] tested many of the ecological and evolutionary hypotheses proposed to explain PF diversity by relating the four diversity measures above to a set of environmental variables chosen to capture ocean temperature, structure, productivity and seasonality (table 1; electronic supplementary materials, figure S2). Their models find support for a combination of ecological and evolutionary models of diversity. Although SST explains the largest portion of diversity in all four diversity measures, observed relationships do not match metabolic theory of ecology or mid-domain model predictions [56]. Historical models are thought to be less significant for PF due to the absence of dispersal limitation in this clade [74]. These results suggest the diversity patterns of PF cannot be explained by any one environmental variable or proposed mechanism but reflect multiple processes acting in concert.

## 3. Descent into the Icehouse

Temporal comparisons are most powerful in testing macroecological models when the predictive time differs markedly in environmental conditions from that of the initial analysis [23]. Previous work suggests that the richness-temperature relationship in PF has remained constant for 3 Myr, though the diversity patterns have changed alongside climate [71]. Here, we consider more aspects of diversity and environment and attempt to transfer the relationship over a much longer time period. The Eocene (56–34 Ma) had a broadly similar palaeogeographic configuration of the major ocean basins as the Recent, and a phylogenetically and ecomorphologically comparable PF community, but a very different global climate, so provides a strong test of model transferability. By considering time-slices through the Eocene it is possible to interrogate the effects of the cooling trend from Early Eocene conditions of Cenozoic peak warmth through to Late Eocene climates, immediately prior to the onset of the present ‘icehouse’ climate state [75]. The Mid- to Late Eocene saw changes in the distribution of ocean fronts and regions of productivity [76,77], as well as in the ocean nutricline and the structuring of planktonic niches [78,79]. By the Late Eocene, global cooling had produced biogeographically distinct high-latitude communities [80].

### (a) Observed diversity through the Eocene

Eocene PF occupied similar niches to extant species, being globally distributed with ecologies dependent on depth habitat, hydrography and mode of life [81]. Many Eocene taxa have analogues in the Recent, such as the digitate morphology in *Clavigerinella jarvisi* (Eocene) and *Beella digitata* (extant); isotopic analyses indicate a deep-dwelling habit for both species [82]. Some Eocene groups and traits however are no longer represented in the biota. Most species with the ‘muricate’ wall structure, characteristic of the acarininids, went extinct in the Eocene, and this morphology was finally lost in the Oligocene [29]. The *Hantkenina* lineage initially occupied a unique warm deep-water niche [79], which was lost when it migrated permanently into a shallower habitat [83]. Previous studies of Eocene diversity have mainly focused on the global picture (e.g. [30,84]), although Boersma & Premoli Silva [80] analysed site-level data from the Atlantic to suggest the onset of an LDG in species richness by the end of the Eocene.

The Eocene PF assemblage data compiled for this project were obtained from a range of sources and have never previously been collated in one place (electronic supplementary material). The basis of the data was the NEPTUNE dataset, a repository of records from the ocean drilling programmes [85,86]. This dataset is now 15 years out of date, so it was supplemented by an extensive literature search including more recent drilling programme publications. The taxonomy of Eocene PF, including full synonymy lists, was revised by Pearson *et al.* [81], and subsequently combined into a look-up table to ensure taxonomic consistency (as used in [29]). Where possible the most recent calibrations of PF zones [46] were used to date the samples. Palaeolocations of sites were calculated using the Getech PlC plate model (following [87]) which determines locations at the time at which samples were deposited. This plate reconstruction is consistent with that used for the environmental data from the climate models described in the next section. Sites that showed high levels of dissolution were excluded to prevent systematic bias [28,88], as were sites where only a fraction of the species were identified, as occurs where the primary purpose was biostratigraphy. Where there were multiple estimates of diversity for the same site within a time interval, the mean diversity observed at a site was chosen to represent the total diversity. (Similar results are obtained if the maximum value is used; not shown). The complete dataset contains 78 sites with a reasonable spatial coverage (electronic supplementary material, figures S3 and S4). To assess latitudinal gradients, the Eocene was divided into three time intervals: Early (56–47.8 Ma), Middle (47.8–38 Ma) and Late (38–33.9 Ma).

We calculated the same four diversity metrics for each site as in the Recent, except that in-sample species richness was used in place of rarefaction-based richness. Both in the Recent and the Eocene, some studies report data as percentage abundance rather than counts of individual. However, the Eocene dataset is too small to include only studies with count data. Where it could be calculated, rarefied richness was strongly correlated with, though usually lower than, observed in-sample richness (linear model: *r*^2^ = 0.69, *n* = 158, *p* < 10^–15^; electronic supplementary material, figure S5).

**Figure 2.**
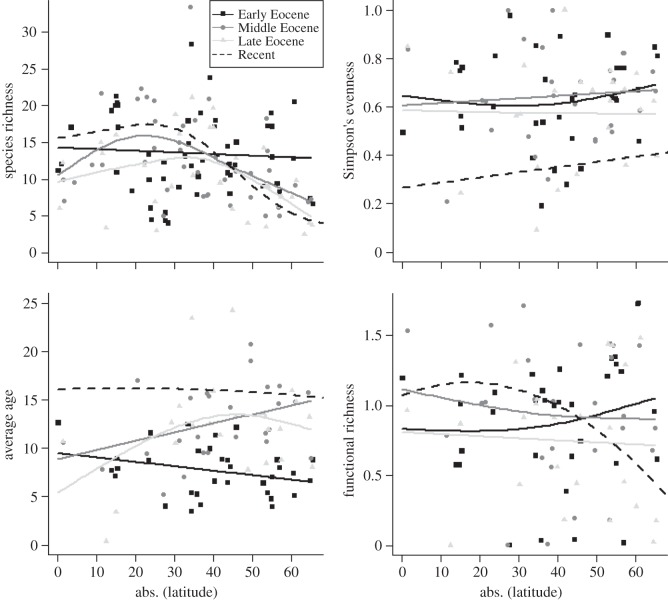
Latitudinal trends in diversity of macroperforate PF through the Eocene; the Recent is added for comparison. GAM smooths are used to highlight the general trends for each time period.

Generalized additive models (GAMs) [89] of assemblage diversity with latitude in each sub-epoch show that the LDG developed during the Eocene (figure 2; electronic supplementary material, figure S6). The significance of these diversity changes does not depend on any individual site (electronic supplementary material, table S1). The gradient changed shape significantly through this time period. Similar to today, Middle and Late Eocene diversity peaked at 20°–30° latitude. Equatorial species richness may have been lower than in the present day, although equatorial sites in the Early Eocene are sparse. The Simpson's evenness gradient did not change significantly through the Eocene, although it was consistently higher than in the present day, implying less dominance by a few species. Functional richness did not change significantly through the Eocene (figure 2). High-latitude functional richness dropped slightly from the Early to the Middle Eocene, indicating loss of some functional groups, whether through extinction or range shift. Despite the drop in species richness at lower latitudes in the Mid–Late Eocene there is no associated drop in the functional richness. This disconnect implies that the LDG is not solely driven by niche availability.

MEA changed significantly through the Eocene, developing a latitudinal gradient. This change was driven by an ageing of high-latitude assemblages, suggesting new species were less able to enter, persist in or dominate these communities. Low-latitude assemblages had similar average ages through time, despite the ageing of the clade as a whole, implying the loss of older species and/or the gain of new ones. The Early Cenozoic rebound from the Cretaceous/Palaeogene mass extinction sets an upper limit on MEA that increases over time. Periods with higher speciation rates, such as the Palaeocene/Eocene boundary and the Mid-Eocene [30], reduce global average MEA estimates.

### (b) Predicted diversity in the Eocene

Testing the transferability of the Recent macroecological models of PF diversity requires estimates of Eocene palaeoenvironment. These are combined with the Recent diversity–environment models to generate predicted diversity values for comparison with the Eocene assemblage data. In this transfer we focus only on the richness–environment model, as richness has the strongest relationship with environment in the Recent (pseudo-*r*^2^ = 0.89; see [56]). Eocene environmental estimates are inevitably far less certain than modern observational data in both accuracy and spatial coverage. Proxy-based estimates, for example, of SST (e.g. [90,91]), have sparse spatial coverage and so are insufficient for spatially explicit global models, although we qualitatively compare trends inferred from these estimates with observed richness data. For spatially explicit global estimates—maps of predicted diversity—we instead use output from an Atmosphere-Ocean General Circulation Model (AOGCM), which provides global coverage of a range of variables. Specifically, we use the HadCM3 L model, as used in Lunt *et al.* [92] and Inglis *et al.* [87]. This model has a spatial resolution of 2.5° by 3.75° (latitude and longitude, respectively), with 19 atmospheric and 20 oceanic levels. For further information on these models, see Inglis *et al.* [87] and Lunt *et al.* [93]. The model has been evaluated against proxy data [87,90].

We considered two suites of simulations from the AOGCM which represent two potential long-term drivers for Eocene climatic cooling: (i) global CO_2_ decline and (ii) plate tectonic changes to oceanic gateways. The first suite—the CO_2_ suite—held palaeogeography constant (in an Early Eocene configuration), altering only the CO_2_ concentrations (Early Eocene—1680 ppmv, Middle Eocene—1120 ppmv, Late Eocene—560 ppmv [92]). The second suite—the tectonic suite—kept CO_2_ levels at 1120 ppmv throughout while changing the land-mass configurations (Early Eocene—Ypresian, Middle Eocene—Bartonian, Late Eocene—Priabonian [87]). These simulations allow us to investigate the individual impact of CO_2_ and tectonic change on species richness. Unfortunately, model simulations which combine both CO_2_ and tectonic change through the Eocene are currently unavailable.

The predicted species richness for each suite of simulations was calculated by combining the palaeoenvironmental data with an adapted version of the richness–environment model developed for the Recent [56]. The list of variables included in this adapted statistical model can be found in table 1; they are the same as the Recent except that productivity and oxygen stress are not predicted in the AOGCM, or otherwise available for the Eocene, so are omitted. The oceanic water mass is also excluded as it is not comparable between the two time periods. GAMs were used rather than spatial autoregressive models, as the former are less likely to give extreme values when extrapolating beyond the range of the data used to fit the model. To mitigate spatial autocorrelation in the GAMs, a two-dimensional smooth of latitude and longitude was included [89]. All statistical modelling analyses were performed in R v. 3.0.3 [94].

Richness was estimated for each grid cell of the environmental data in each time period. The goodness-of-fit between observations and the model predictions for the corresponding grid cells was quantified using the RMSE, the average absolute departure of points from the fitted values. For each simulation's predictions, the Akaike information criterion (AIC) of how much the observed differed from the predicted diversity was calculated [95]. We compared the goodness-of-fit of the simulations in the CO_2_ and the tectonic suites, using ΔAIC to determine whether the richness data provide stronger support for either simulation. Models with ΔAIC > 4 are taken as having ‘considerably less’ support than the minimum-AIC model [96]. As SST is the most important single correlate of species richness in the Recent ([16]; electronic supplementary material, figure S2), temperature estimates are likely to have the greatest influence on the richness estimates. To test whether temperature by itself is sufficient to explain the observed richness, a similar GAM containing only temperature was estimated from the Recent data and used to estimate Eocene richness. The RMSE values for this statistical model were also calculated for comparison with the full model.

**Figure 3.**
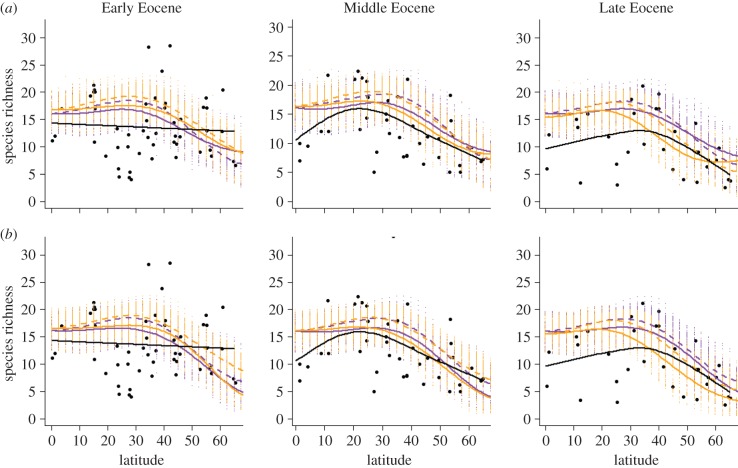
Observed and predicted Eocene diversity. (*a*) Predictions from the full GAM. (*b*) Predictions from a temperature only model. Black points/lines are the observed data. Purple (tectonic) and orange (CO_2_) points are the model predictions for individual grid cells, with smooths to show the latitudinal trend (continuous lines: Northern Hemisphere; dashed lines: Southern Hemisphere).

Figure 3 compares the predicted and the observed Eocene species richness; table 2 gives the corresponding RMSE and ΔAIC values. These results suggest that the tectonic and CO_2_ simulations differ little in their predictions for the latitudinal patterns of richness. The fit at high latitudes is best by the end of the Eocene; at low latitudes, the Middle Eocene produces a slightly better fit (table 2). However, the overall shape is correctly predicted for the Middle and Late Eocene, but not for the Early Eocene. The tectonic simulations fit significantly better in the Early Eocene, but the CO_2_ simulations provide the markedly better fit by the end of the Eocene. Re-running the analysis using only the predicted relationship between temperature and richness produces only marginally better fit throughout the Eocene (table 2), with the Early Eocene shape still incorrect (figure 3).

**Table 2.** RMSE, with ΔAIC reported in brackets, of the different models in this analysis.

diversity measure	model	Early Eocene	Middle Eocene	Late Eocene
mean species richness—full	tectonic	7.04 (0)	5.93 (0)	6.27 (+7.69)
	CO_2_	7.65 (+28.0)	6.72 (+2.60)	5.41 (0)
mean species richness—temperature	tectonic	5.76 (0)	5.39 (0)	5.25 (+9.91)
	CO_2_	6.18 (+23.3)	6.16 (+0.21)	5.01 (0)

## 4. Possible reasons for the mismatch between predicted and observed diversity

The lack of fit between assemblage data and model predictions suggests that the relationship between foraminiferal richness and environmental drivers observed today transfers imperfectly to the Eocene, especially the Early Eocene (figure 3 and table 2). There are three possible reasons for this mismatch. First, the AOGCM estimates of the palaeoenvironmental variables used in this analysis could be incorrect. If so, even a statistical model that correctly captured the environment–richness relationship would appear to fail in the Eocene. Second, the variables included in the model for the Recent could have failed to capture the true drivers of diversity, so any attempt to transfer into a very different past would automatically fail. Third, the relationship between environment and richness could have fundamentally changed since the Eocene. In this case, however correct the statistical model is in the Recent, it would not predict the past accurately. We consider each of these possibilities in turn.

### (a) Estimates of Eocene environment

In the Early Eocene, the assemblage data suggest that the LDG is basically flat from the Equator to ±60°, whereas the model predicts a strong gradient in richness. This prediction arises from the strong latitudinal gradient in temperature present even in the Early Eocene in both the simulations (electronic supplementary material, figure S7). Proxy temperature records, by contrast, indicate a weaker [90,91] or minimal [87] Early Eocene temperature gradient, which is inferred to have strengthened through the Eocene [87,97,98]. The proxy-based climate estimates are qualitatively more compatible with the observed richness than are the (spatially explicit) climate models we have used. Recent efforts to resolve this proxy-climate model mismatch, based around the choice of parameters associated with clouds and aerosols, are promising [99–101] and have the potential to resolve the offsets in observed and predicted richness patterns. However, it is outside the scope of this study to test this possibility.

### (b) Explanatory variables for the global diversity model

Several environmental variables—oxygen stress, productivity and ocean—had to be excluded from our analysis, despite having been shown to be significant predictors of Recent diversity ([56]; electronic supplementary material, figure S2). Levels of oxygen stress in the Eocene are not clear, although there may have been a more pronounced oxygen minimum in the upper water column due to faster microbial respiration of sinking organic matter [78,102]. There is some evidence that productivity was high during parts of the Eocene [103,104]. The differences between Eocene and Recent in the tectonic plate position and the locations of ocean gateways mean that ocean effects on diversity are likely to have altered. Again we cannot test the significance of these missing variables, as they are not currently available from the global climate models.

Many environmental variables are strongly correlated with latitude, and consequently, with each other (indeed, this is essentially why the driving mechanisms for the LDG remains a topic of debate [15]). This correlation makes identifying the true explanatory variables difficult. For example, temperature is known to be important, but in the present day there are strong correlations between mean annual, mean summer and maximum temperature at a site. Each could be used equally well in Recent statistical models, although any one of them might be the variable actually driving richness. If the correlations between these variables have changed, then choosing the wrong variable could lead to a statistical model with a poorer fit in the Eocene. It is very challenging to identify these ‘true’ variables without a deeper understanding of the ecological response of the foraminifera to their environment.

### (c) Diversity–environment relationships

The third explanation suggests the response variable, rather than the explanatory variables, does not fully capture the underlying diversity. Although species richness is often used as a shorthand for biodiversity [15,105], no single number can adequately capture all facets of biodiversity [106]. Diversity measures are often expected to be intercorrelated [107], but the correlations can vary among study systems. For example, although functional diversity usually rises with species richness, there are exceptions [108,109]. Species richness is often the simplest metric to measure, but may not be the most informative for understanding community structure. The mechanisms that structure communities act upon the ecological similarities, differences and interactions between species, not on numbers of species [110], and the relationships between species richness and other diversity metrics could change through time or space as well as differing among taxonomic groups or ecological guilds. If the relationship between species richness and ‘true’ diversity (i.e. the idealized measure of diversity that is determined by the environmental drivers) changes, a model relating richness to environment will not transfer, even if the underlying relationships between ‘true’ diversity and environment are unchanged.

To explore this possibility, we undertook a multivariate analysis of six diversity measures, to characterize their intercorrelations in the Recent and the combined Eocene dataset. These diversity measures are the four already described (species richness, Simpson's evenness, MEA and functional richness) and two closely related measures (mean morphological age and Simpson's diversity). The mean morphological age is the (abundance-weighted) average age of the morphospecies present in an assemblage. As several of the variables were non-normal, we used a robust principal components analysis (rPCA), which scales the data using the median and the median absolute deviation, not the mean and standard deviation [111,112]. To test whether these two rPCAs differ significantly, we compare the observed PCA similarity [113] to a null distribution obtained of PCA similarity scores obtained from 1000 randomization trials in which the assemblages were randomly divided into two groups having the observed sample sizes.

**Figure 4.**
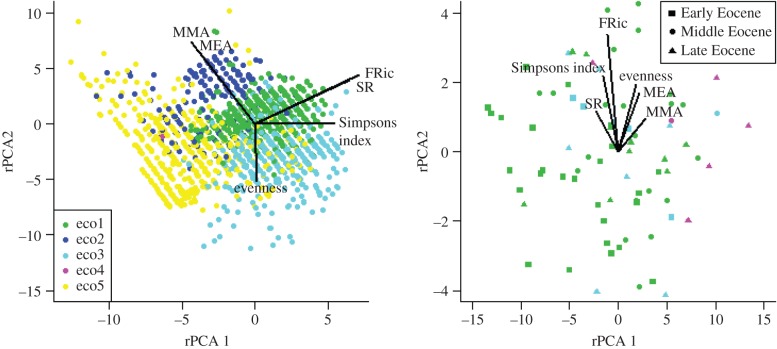
Recent and Eocene rPCA relationships, coloured by the dominant ecogroup [29] at a site. Eco1, open-ocean mixed-layer tropical/subtropical, with symbionts; eco2, open-ocean mixed-layer tropical/subtropical, without symbionts; eco3, open-ocean thermocline; eco4, open-ocean sub-thermocline; eco5, high-latitude. Diversity measures: SR, species richness; FRic, functional richness; MEA, mean evolutionary age; MMA, mean morphological age.

The first three axes of the Recent rPCA explain 94% of the variance; for the Eocene, the first three explain 83% of the variance. The rPCA results for the Recent and the Eocene (figure 4; electronic supplementary material, figure S8) show the relationship between different aspects of diversity have indeed changed through time. For example, species richness is strongly correlated with functional richness in the Recent but not in the Eocene. The differences are more pronounced for rPCA2 and rPCA3 than for rPCA1. Assemblages dominated by the same ecogroup are strongly clustered in the Recent, but not in the Eocene. The randomization test suggests that the Eocene and Recent rPCA structures are near-significantly more different than would be expected by chance (PCAsim = 0.87, significance = 0.071). If we further split the data into each individual time period, there is a suggestion that the diversity structure becomes more similar to the Recent through the Eocene (electronic supplementary material, table S2); however, the Eocene datasets are too small to reliably assess the significance of these relationships. A consequence of this apparent change in diversity structure is that, if the drivers of diversity in fact control some facet of diversity other than species richness, then observed species richness could be only a secondary response: the most commonly used measure of biodiversity may be an epiphenomenon, albeit an extremely useful one for making comparisons within the domain where the biodiversity dimensionality is broadly constant.

## 5. Conclusion

Planktonic foraminifera are unusual in allowing the study of how assemblage diversity changes over large spatial and temporal scales. Our analyses identify the ‘Descent into the Icehouse’—the global cooling trend through the Eocene—as a key period for the development of the LDG within the clade. This analysis suggests the richness–environment relationships seen in the Recent first appeared in the Mid–Late Eocene, even though the modern PF faunas and provinces did not develop until the Miocene [30,114]. Our results highlight the Eocene itself, rather than the Eocene–Oligocene boundary, as the time during which the current relationships between environmental drivers and diversity—at least in this clade—became established. An alternative way of viewing the similarity through time in the richness–environment relationship is that PF diversity may respond relatively quickly to environmental change (although this conclusion is tentative pending a comprehensive analysis of assemblage diversity through the entire Cenozoic). Their lack of dispersal limitation (e.g. [39]) could be key to this apparently rapid response. In groups with more limited dispersal, the spatial and temporal patterns of speciation and extinction (e.g. [5]) may be more important in structuring diversity gradients.

Richness–environment relationships appear to have been different in the Early Eocene, but we cannot distinguish among several possible explanations: poor estimates of environmental variables (suggested by the mismatch between proxies and general circulation models), missing environmental variables, or a fundamental change in the structure of Eocene diversity (shown by the rPCA results and suggested by the extinctions that occur though this time period). However, the similar pattern of high-latitude mismatches when comparing modelled richness with observed richness, and climate model output with climate proxy data suggest that improved models of high-latitude greenhouse climates may be critical to resolving these patterns of biodiversity and climate.

## Supplementary Material

Supplementary bibliography

## Supplementary Material

Supplementary figures and tables

## Data accessibility

Eocene diversity data: NHM databank (http://dx.doi.org/10.5519/0036305). For more information, see the electronic supplementary material. Recent diversity data available from MARGO: http://doi.pangaea.de/10.1594/PANGAEA.841194.
