# Supplementary material for: The impact of Cenozoic cooling on assemblage diversity in planktonic foraminifera
Source: Philos Trans R Soc Lond B Biol Sci. 2016 Apr 5;371(1691):20150224. doi: 10.1098/rstb.2015.0224 (PMC4810817; doi:10.1098/rstb.2015.0224)
Supplement: Supplementary bibliography [file rstb20150224supp1.docx]

***
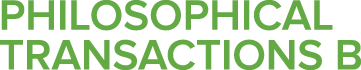
****Phil. Trans. R. Soc. B.* doi: 10.1098/rstb.2015.0224

The impact of Cenozoic cooling on assemblage diversity in planktonic foraminifera

Isabel S. Fenton, Paul N. Pearson, Tom Dunkley Jones, Alexander Farnsworth, Daniel J. Lunt, Paul Markwick and Andy Purvis

**Data sources**

The basis of the data was the NEPTUNE dataset that was compiled as a subset of DSDP and ODP records (Lazarus, 1994; Spencer-Cervato, 1999). This dataset was supplemented by a Web of Science search using the key terms “plankt*” and “foraminifera”, and “Eocene” and a large print collection of papers containing information on Eocene foraminifera. The data is available at <http://dx.doi.org/10.5519/0036305>.

The NEPTUNE dataset was supplemented by the following sources:

Arenillas, I., Molina, E. & Schmitz, B. (1999) Planktic foraminiferal and 13C isotopic changes across the Paleocene/Eocene boundary at Possagno (Italy). *International Journal of Earth Sciences*, **88**, 352-364.

Berggren, W. (1992) Paleogene planktonic foraminifer magnetobiostratigraphy of the southern Kerguelen Plateau (Sites 747–749). In: *Proceedings of the Ocean Drilling Program, Scientific Results* eds. S.W. Wise Jr., R. Schlich, A.A. Palmer Julson, M.-P. Aubry, W.A. Berggren, P.R. Bitschene, N.A. Blackburn, J. Breza, M.F. Coffin, D.M. Harwood, F. Heider, M.A. Holmes, W.R. Howard, H. Inokuchi, K. Kelts, D.B. Lazarus, A. Mackensen, T. Maruyama, M. Munschy, E. Pratson, P.Q. Quilty, F. Rack, V.J.M. Salters, J.H. Sevigny, M. Storey, A. Takemura, D.K. Watkins, H. Whitechurch and J. Zachos), pp. 551-568

Berggren, W.A., van den Borch, C. & Sclater, J.G. (2005) Planktic foraminifera abundance of Hole 22-214. In, PANGAEA.

Boersma, A., Moore, T.C. & Rabinowitz, P.D. (2005) Foraminifera abundance of Hole 74-529. In, PANGAEA.

Canudo, J.I. & Molina, E. (1992) Planktic foraminiferal faunal turnover and bio-chronostratigraphy of the Paleocene-Eocene boundary at Zumaya, northern Spain. *Revista de la Sociedad Geologica de Espana*, **5**, 145-157.

Egger, H., Roegl, F. & Wagreich, M. (2004) Biostratigraphy and facies of Paleogene deep-water deposits at Gams (Gosau Group, Austria). *Annalen des Naturhistorischen Museums in Wien A Mineralogie Petrologie Geologie Palaeontologie Archaeozoologie Anthropologie Praehistorie*, **106A**, 281-307.

Egger, H., Homayoun, M., Huber, H., Rögl, F. & Schmitz, B. (2005) Early Eocene climatic, volcanic, and biotic events in the northwestern Tethyan Untersberg section, Austria. *Palaeogeography, Palaeoclimatology, Palaeoecology*, **217**, 243-264.

Galeotti, S., Coccioni, R. & Gersonde, R. (2002) Middle Eocene–Early Pliocene Subantarctic planktic foraminiferal biostratigraphy of Site 1090, Agulhas Ridge. *Marine Micropaleontology*, **45**, 357-381.

Gebhardt, H., Adekeye, O.A. & Akande, S.O. (2010) Late Paleocene to initial Eocene thermal maximum (IETM) foraminiferal biostratigraphy and paleoecology of the Dahomey Basin, Southwestern Nigeria. *Jahrbuch der Geologischen Bundesanstalt*, **150**, 407-419.

Gebhardt, H., ĆORIĆ, S., DARGA, R., BRIGUGLIO, A., SCHENK, B., WERNER, W., ANDERSEN, N. & SAMES, B. (2013) Middle to Late Eocene paleoenvironmental changes in a marine transgressive sequence from the northern Tethyan margin (Adelholzen, Germany). *Austrian Journal of Earth Sciences*, **106/2**, 45-72.

Gervais, E. (1996) Cretaceous to Quaternary planktonic foraminiferal biostratigraphy of the Iberia Abyssal Plain. In: *Proceedings of the Ocean Drilling Program, Scientific Results, College Station, TX (Ocean Drilling Program)* eds. R. Whitmarsh, D. Sawyer, A. Klaus and D. Masson), pp. 165-192

Gohrbandt, K. (1967) Some new planktonic foraminiferal species from the Austrian Eocene. *Micropaleontology*, 319-326.

Hamilton, E.L. & Rex, R.W. (1959) Lower Eocene phosphatized Globergerina ooze from Sylvania Guyot. *U.S. Geological Survey Professional Paper*, **260-W**, 785-798.

Hancock, H.J.L., Chaproniere, G.C., Dickens, G.R. & Henderson, R.A. (2002) Early Palaeogene planktic foraminiferal and carbon isotope stratigraphy, Hole 762C, Exmouth Plateau, northwest Australian margin. *Journal of Micropalaeontology*, **21**, 29-42.

Imam, M.M. (2001) Biostratigraphy of the Upper Cretaceous-Lower Eocene succession in the Bani Walid area, northwest Libya. *Journal of African Earth Sciences*, **33**, 69-89.

Kalia, P. & Kintso, R. (2006) Planktonic foraminifera at the Paleocene/Eocene boundary in the Jaisalmer Basin, Rajasthan, India. *Micropaleontology*, **52**, 521-536.

Loubere, P. (1985) Population diversity of planktonic foraminifera and the stable isotope record across the Eocene Oligocene boundary: Hole 549A. In: *Initial Reports of the Deep Sea Drilling Project*, pp. 557-566

Lu, G. & Keller, G. (1993) The Paleocene-Eocene transition in the Antarctic Indian Ocean: Inference from planktic foraminifera. *Marine Micropaleontology*, **21**, 101-142.

Luciani, V., Negri, A. & Bassi, D. (2002) The Bartonian–Priabonian transition in the Mossano section (Colli Berici, north-eastern Italy): a tentative correlation between calcareous plankton and shallow-water benthic zonations. *Geobios*, **35, Supplement 1**, 140-149.

Luterbacher, H., Hollister, C.D. & Ewing, J.I. (2005a) Planktic foraminifera abundance of Hole 11-108. . In, PANGAEA.

Luterbacher, H., Hollister, C.D. & Ewing, J. (2005b) Planktic foraminifera abundance of Hole 11-98. In, PANGAEA.

Luterbacher, H., Larson, R.L. & Moberly, R. (2005c) Planktic foraminifera abundance of Hole 32-313. In, PANGAEA.

McGowran, B., van den Borch, C. & Sclater, J.G. (2005a) Foraminifera abundance of Hole 22-215. In, PANGAEA.

McGowran, B., van den Borch, C. & Sclater, J.G. (2005b) Foraminifera abundance of Hole 22-213. In, PANGAEA.

Mohiuddin, M.M. & Ogawa, Y. (1996) Middle Eocene to early Oligocene planktonic foraminifers from the micritic limestone beds of the Heguri area, Mineoka Belt, Boso Peninsula, Japan. *Journal of the Geological Society of Japan*, **102**, 611-621.

Nishi, H. & Chaproniere, G.C. (1994) Eocene-Oligocene subtropical planktonic foraminifers at Site 841. In: *Proceedings of the Ocean Drilling Program, Scientific Results, College Station, TX (Ocean Drilling Program)* eds. J.W. Hawkins, L.M. Parson, J.F. Allan, N. Abrahamsen, U. Bednarz, G. Blanc, S.H. Bloomer, R. Bøe, T.R. Bruns, W.B. Bryan, G.C.H. Chaproniere, P.D. Clift, A. Ewart, M.G. Fowler, J.M. Hergt, R.A. Hodkinson, D. Lavoie, J.K. Ledbetter, C.J. Macleod, K. Nilsson, H. Nishi, C.E. Pratt, P.J. Quinterno, R.R. Reynolds, R.G. Rothwell, W.W. Sager, D. Schöps, S. Soakai and M.J. Styzen), pp. 245-266

Nocchi, M., Amici, E. & Premoli Silva, I. (1991) Planktonic foraminiferal biostratigraphy and paleoenvironmental interpretation of Paleogene faunas from the subantarctic transect, Leg 114. In: *Proceedings of the Ocean Drilling Program, Scientific Results, College Station, TX (Ocean Drilling Program)* eds. P.F. Ciesielski, Y. Kristoffersen, B. Clement, J.-P. Blangy, R. Bourrouilh, J.A. Crux, J.M. Fenner, P.N. Froelich, E. Hailwood, D.A. Hodell, M.E. Katz, H.Y. Ling, J. Mienert, D. Muller, C.J. Mwenifumbo, D.C. Nobes, M. Nocchi, D.A. Warnke and F. Westall), pp. 233-279

Nocchi, M., Parisi, G., Monaco, P., Monechi, S. & Madile, M. (1988) Eocene and early Oligocene micropaleontology and paleoenvironments in SE Umbria, Italy. *Palaeogeography, Palaeoclimatology, Palaeoecology*, **67**, 181-244.

Olsson, R.K. (1970) Paleocene Planktonic Foraminiferal Biostratigraphy and Paleozoogeography of New Jersey. *Journal of Paleontology*, **44**, 589-597.

Ortiz, S., Gonzalvo, C., Molina, E., Rodríguez-Tovar, F.J., Uchman, A., Vandenberghe, N. & Zeelmaekers, E. (2008) Palaeoenvironmental turnover across the Ypresian–Lutetian transition at the Agost section, Southastern Spain: In search of a marker event to define the Stratotype for the base of the Lutetian Stage. *Marine Micropaleontology*, **69**, 297-313.

Pälike, H., Lyle, M., Nishi, H., Raffi, I., Gamage, K., Klaus, A. & the Expedition 320/321 Scientists (2010) Site U1333. *Proceedings of the Integrated Ocean Drilling Program*, **320/321**

Pardo, A., Keller, G. & Oberhaensli, H. (1999) Paleoecologic and paleoceanographic evolution of the Tethyan realm during the Paleocene-Eocene transition. *The Journal of Foraminiferal Research*, **29**, 37-57.

Pardo, A., Keller, G., Molina, E. & Canudo, J. (1997) Planktic foraminiferal turnover across the Paleocene-Eocene transition at DSDP Site 401, Bay of Biscay, North Atlantic. *Marine Micropaleontology*, **29**, 129-158.

Petrizzo, M.R. (2007) The onset of the Paleocene–Eocene Thermal Maximum (PETM) at Sites 1209 and 1210 (Shatsky Rise, Pacific Ocean) as recorded by planktonic foraminifera. *Marine Micropaleontology*, **63**, 187-200.

Petrizzo, M.R., Premoli Silva, I. & Ferrari, P. (2005) Data report: Paleogene planktonic foraminifer biostratigraphy, ODP Leg 198 holes 1209A, 1210A, and 1211A (Shatsky Rise, northwest Pacific Ocean). In: *Proceedings of the Ocean Drilling Program, Scientific Results, College Station, TX (Ocean Drilling Program)* eds. T. Bralower, I. Premoli Silva and M. Malone), pp. 1-56

Poore, R.Z. & Brabb, E.E. (1977) Eocene and Oligocene planktonic foraminifera from the Upper Butano sandstone and type San Lorenzo Formation, Santa Cruz Mountains, California. *Journal foram Res*, **7**, 249-272,illust.

Saito, T. (1962) Eocene planktonic foraminifera from Hahajima (Hillsborough Island). *Transactions and proceedings of the Palaeontological Society of Japan*, **45**, 209-225.

Samanta, B.K. (1969) Eocene planktonic Foraminifera from the Garo Hills, Assam, India. *Micropaleontology*, **15**, 325-350.

Samanta, B.K. (1970) Middle Eocene planktonic foraminifera from Lakhpat, Cutch, Western India. *Micropaleontology*, **16**, 185-215.

Samanta, B.K. (1973) Planktonic Foraminifera from the Palaeocene-Eocene Succession in the Rakhi Nala, Sulaiman Range, Pakistan. *Bulletin of the British Museum (Natural History), Geology*, **22**, 421-482.

Snyder, S.W. & Waters, V.J. (1985) Cenozoic planktonic foraminiferal biostratigraphy of the Goban Spur Region, Deep Sea Drilling Project Leg 80. In: *Initial Reports of the Deep Sea Drilling Project* eds. P. De Graciansky, C. Poag, R. Cunningham Jr., P. Loubere, D.G. Masson, J.M. Mazzullo, L. Montadert, C. Müller, K. Otsuka, L. Reynolds, J. Sigal, S. Snyder, H.A. Townsend, S.P. Vaos and D. Waples), pp. 439-472, Washington (U.S. Gov. Printing Office).

Spezzaferri, S., Basso, D. & Coccioni, R. (2002) Late Eocene planktonic foraminiferal response to an extraterrestrial impact at Massignano GSSP (northeastern Appennines, Italy). *The Journal of Foraminiferal Research*, **32**, 188-199.

Sztrákos, K. (2005) Les foraminifères du Paléocène et de l'Éocène basal du sillon nord-pyrénéen (Aquitaine, France). *Revue de Micropaléontologie*, **48**, 175-236.

Toumarkine, M., Bolli, H.M. & Ryan, W.B.F. (2005a) Planktic foraminifera abundance of Hole 40-360. In, PANGAEA.

Toumarkine, M., Bolli, H.M. & Ryan, W.B.F. (2005b) Planktic foraminifera abundance of Hole 40-361. In, PANGAEA.

Toumarkine, M., Bolli, H.M. & Ryan, W.B.F. (2005c) Planktic foraminifera abundance of Hole 40-364. In, PANGAEA.

Wade, B.S. & Pearson, P.N. (2008) Planktonic foraminiferal turnover, diversity fluctuations and geochemical signals across the Eocene/Oligocene boundary in Tanzania. *Marine Micropaleontology*, **68**, 244-255.

Warraich, M.Y. & Natori, H. (1997) Geology and planktonic foraminiferal biostratigraphy of the Paleocene-Eocene succession of the Zinda Pir section, Sulaiman Range, Southern Indus Basin, Pakistan. *Bulletin of the Geological Survey of Japan*, **48**, 595-630.

Warraich, M.Y. & Nishi, H. (2003) Eocene planktic foraminiferal biostratigraphy of the Sulaiman Range, Indus Basin, Pakistan. *The Journal of Foraminiferal Research*, **33**, 219-236.

Warraich, M.Y., Ogasawara, K. & Nishi, H. (2000) Late Paleocene to Early Eocene planktic foraminiferal biostratigraphy of the Dungan Formation, Sulaiman Range, central Pakistan. *Paleontological Research*, **4**, 275-301.
