# Supplementary material for: The impact of Cenozoic cooling on assemblage diversity in planktonic foraminifera
Source: Philos Trans R Soc Lond B Biol Sci. 2016 Apr 5;371(1691):20150224. doi: 10.1098/rstb.2015.0224 (PMC4810817; doi:10.1098/rstb.2015.0224)
Supplement: Supplementary figures and tables [file rstb20150224supp2.pdf]

PHILOSOPHICAL TRANSACTIONS B

Phil. Trans. R. Soc. B.

doi: 10.1098/rstb.2015.0224

The impact of Cenozoic cooling on assemblage diversity in planktonic foraminifera

Isabel S. Fenton, Paul N. Pearson, Tom Dunkley Jones, Alexander Farnsworth, Daniel J. Lunt, Paul Markwick and Andy Purvis

Supplementary information

	Species richness	Species richness 2	Simpson's evenness	Mean Evolutionary Age	Functional richness
Full model	0.0317	0.0164	0.9127	0.00445	0.432
Min.	0.0081	0.0043	0.5407	0.0000313	0.2104
Median	0.0437	0.0171	0.9127	0.00445	0.4319
Mean	0.0416	0.0170	0.8981	0.00498	0.4366
Max.	0.0719	0.0237	0.9965	0.0272	0.6344

Supplementary Table 1. P-values from testing whether the diversity-latitude relationship differs significantly between the three Eocene time periods. These values are calculated by comparing a GAM of diversity against latitude where each time interval (i.e. early Eocene, middle Eocene, late Eocene) has a different intercept and slope, with a GAM where the intercepts differ but the slopes are fixed. For all the diversity columns except the second all time-intervals are treated separately. The second species richness column gives the results of combining the middle and late Eocene slopes. For the rows, the full model shows the results with the entire dataset. The remaining rows relate to jackknifing, i.e. testing the reliance of the results on any one point. They show the significance of this comparison when each dataset is excluded in turn. P-values <0.05 imply that the more complex model (i.e. separate slopes and intercepts) is necessary to capture the complexity of the data.

	Early	Middle	Late	Recent
Early	x	0.831 (0.212)	0.838 (0.265)	0.816 (0.075)
Middle	x	x	0.935 (0.938)	0.832 (0.227)
Late	x	x	x	0.856 (0.370)

Supplementary Table 2. PCA similarity scores for each of the paired comparisons of the periods. The numbers in brackets are the significance of these differences.

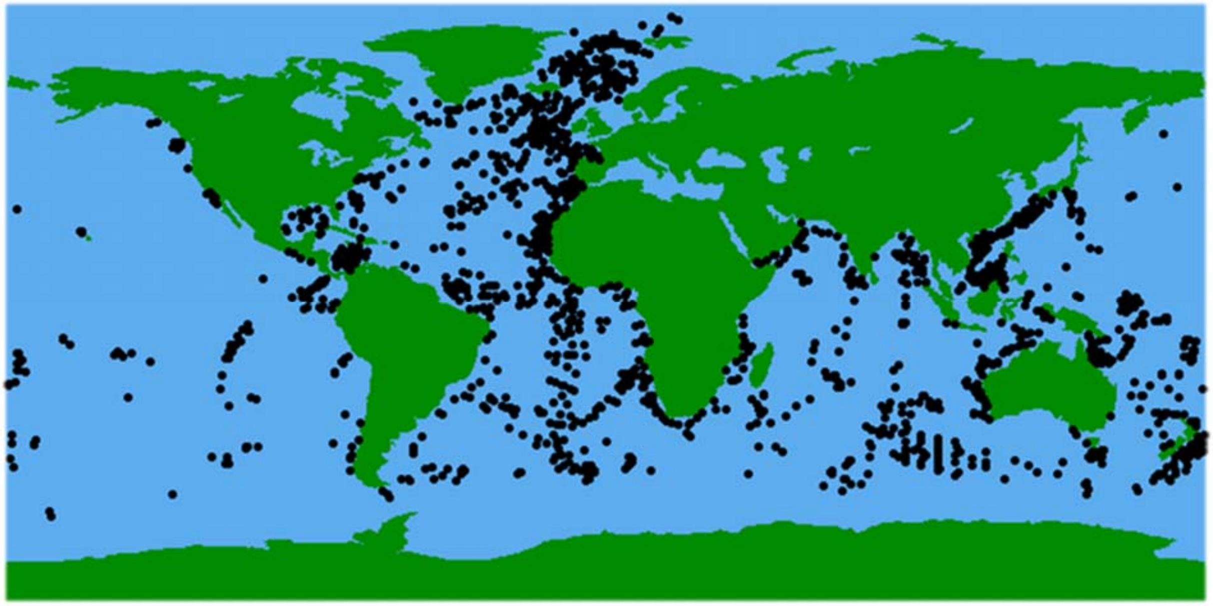

Supplementary Figure 1. Distribution of sites for the extant diversity. Data from MARGO [48].

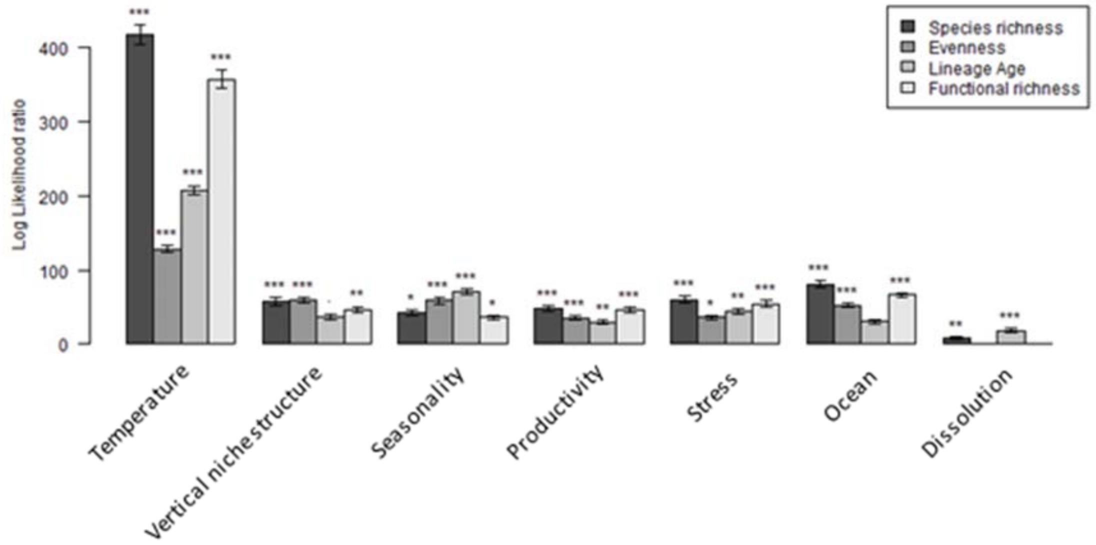

Supplementary Figure 2. A comparison of the relative explanatory power of the groups of explanatory variables for the models of the four response variables. Error bars show 1sd and represent the variation associated with removing the replication within each 1 degree square. Figure from [56].

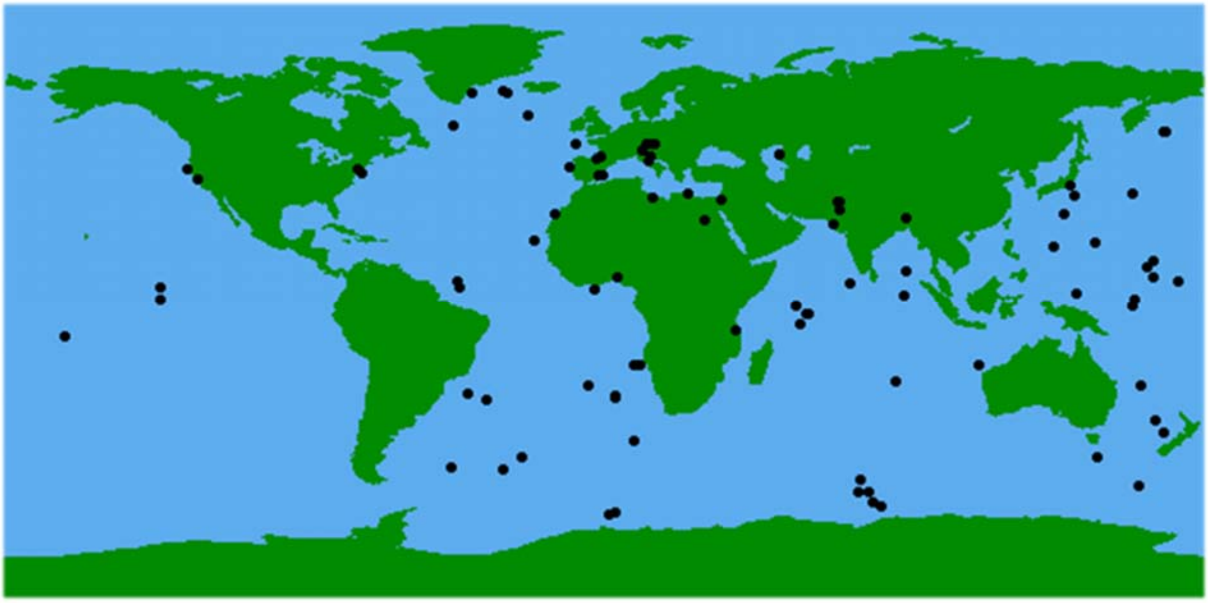

Supplementary Figure 3. Distribution of sites for the Eocene diversity. Data collated from the literature for this study.

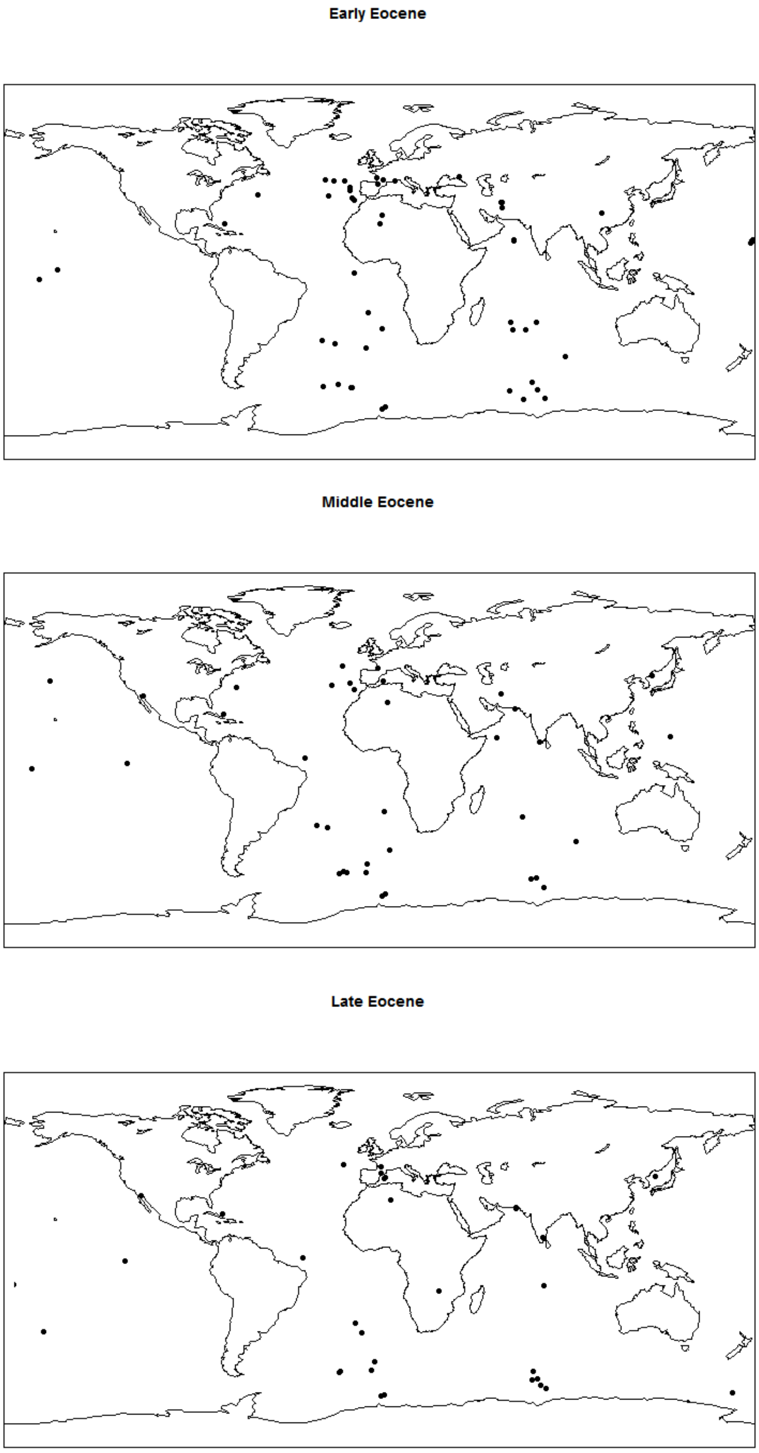

Supplementary Figure 4. The palaeolocations of sites for each period (cf. Supplementary Figure 3 which shows the present day locations). Present day outlines are added for ease of interpretation. Recent has 2389 sites; Early Eocene has 32 sites; Middle Eocene has 26 sites; Late Eocene has 23 sites.

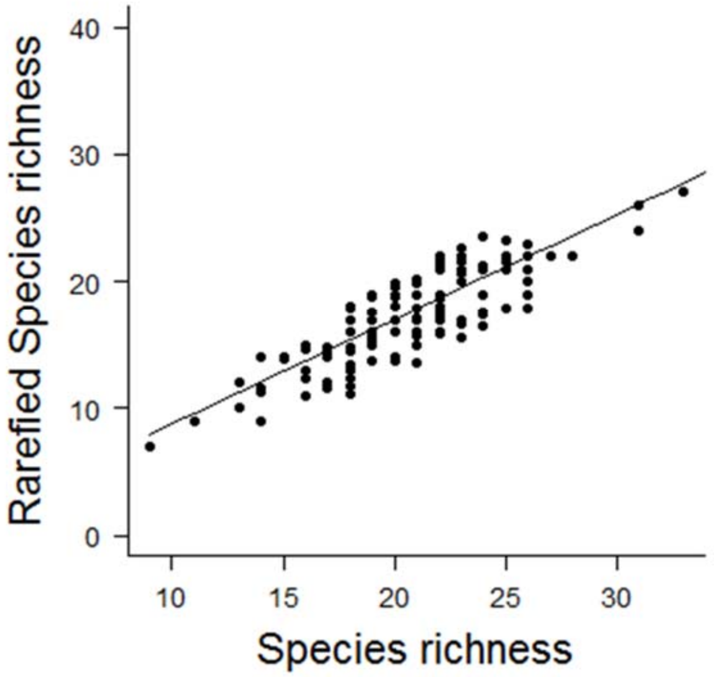

Supplementary Figure 5. Comparison of rarefied (black) and normal species richness for those Eocene sites with abundance counts. Rarefied richness was calculated for 275 individuals. The lines join the species richness and rarefied richness for sites.

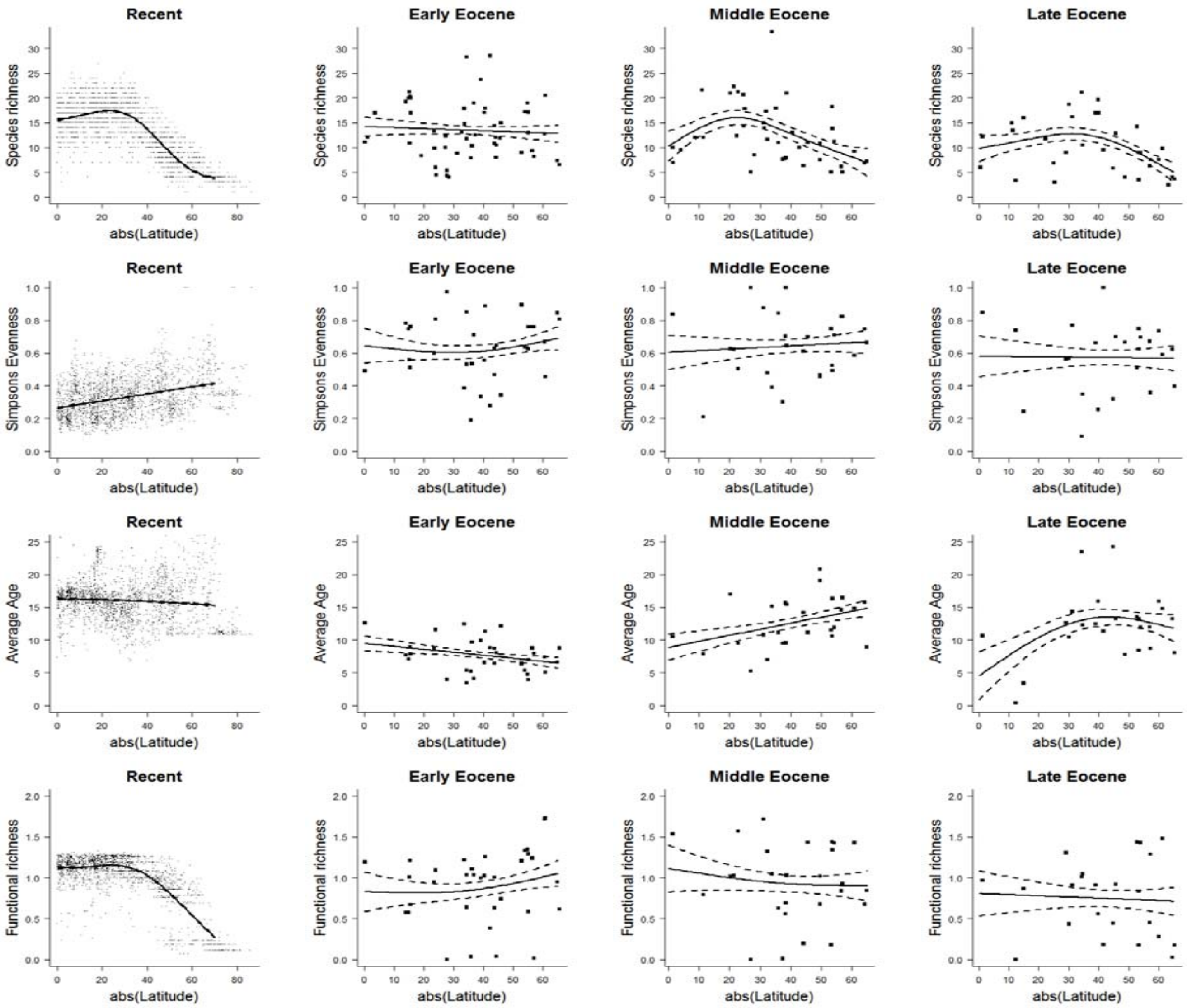

Supplementary Figure 6. The GAM smooths and the data points for the diversity measures both in the Eocene and the Recent (see Figure 2). The dashed lines represent the standard errors.

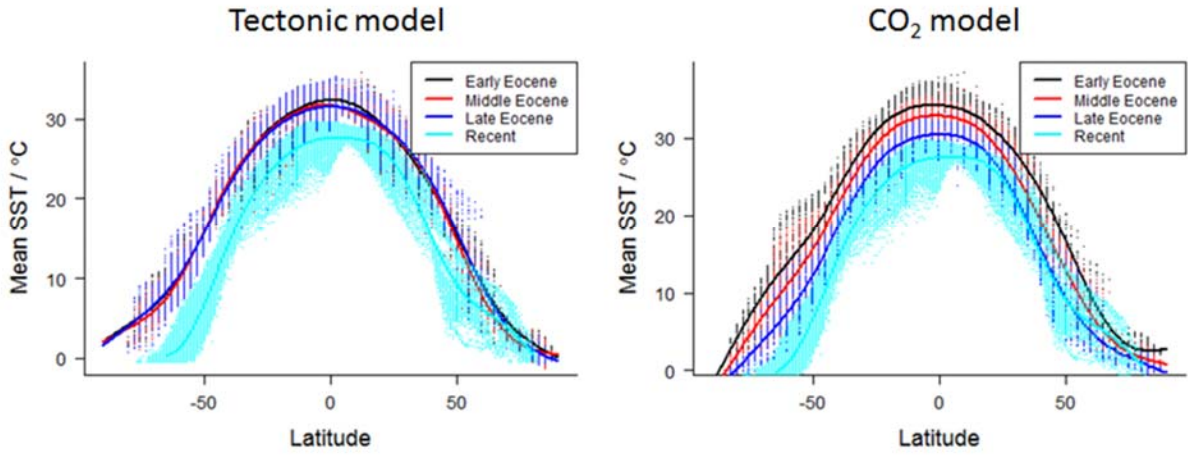

Supplementary Figure 7. The sea-surface temperature gradient with latitude for the Eocene and the Recent, for the two different climate models tested.

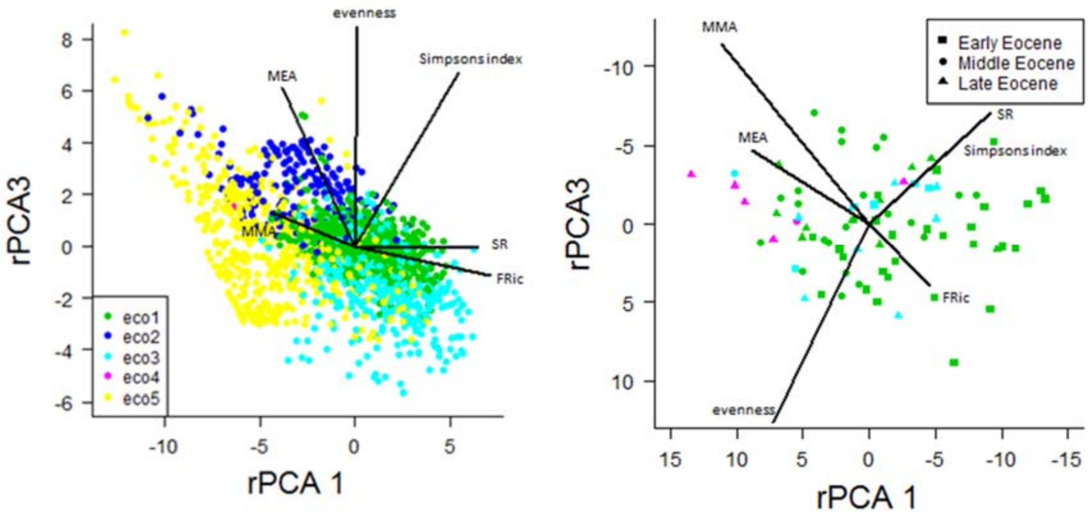

Supplementary Figure 8. Recent and Eocene rPCA relationships, axes 1 and 3. The colours represent the dominant ecogroup [29] at a site: eco1, open ocean mixed-layer tropical/ subtropical, with symbionts; eco2, open ocean mixed-layer tropical/ subtropical, without symbionts; eco3, open ocean thermocline; eco4, open ocean sub-thermocline; eco5, high-latitude.

© The Authors under the terms of the Creative Commons Attribution License http://creativecommons.org/licenses/by/3.0/, which permits unrestricted use, provided the original author and source are credited.
